# Long-Term Outcomes of Drug-Refractory Atrial Fibrillation After Atrioventricular Node Ablation Combined with Implantation of a Cardiac Resynchronization Therapy Defibrillator (CRT-D)

**DOI:** 10.3390/jcm14248938

**Published:** 2025-12-18

**Authors:** Bagdat A. Akhyt, Salim F. Berkinbaev, Natalya G. Lozhkina, Sergey N. Artemenko, Nikolay Yu. Zyatkov, Olga I. Krivorotko, Kulzida M. Koshumbayeva, Marat O. Pashimov, Rustem M. Tuleutayev, Elmira B. Kultanova

**Affiliations:** 1 JSC “Research Institute of Cardiology and Internal Medicine”, Almaty 050000, Kazakhstan; bagdat.ahyt@mail.ru (B.A.A.); kulzidamk@mail.ru (K.M.K.); rustemtuleutayev@gmail.com (R.M.T.); dsrm.ricid@gmail.com (E.B.K.); 2 Department of Cardiology, Faculty of Postgraduate Education, National Joint-Stock Company “Kazakh National Medical University Named After S.D. Asfendiyarov”, Almaty 050000, Kazakhstan; berkinbaev.s@kaznmu.kz; 3 Federal Research Center for Fundamental and Translational Medicine, Novosibirsk 630117, Russia; lozhkina.n@mail.ru (N.G.L.); artem_sn@rambler.ru (S.N.A.); 4 Federal State Autonomous Educational Institution of Higher Professional Education Novosibirsk National Research State University, Novosibirsk 630090, Russia; 5 S.L. Sobolev Institute of Mathematics, Siberian Branch, Russian Academy of Sciences, Novosibirsk 630090, Russia; n.y.zyatkov@math.nsc.ru (N.Y.Z.); krivorotko.olya@mail.ru (O.I.K.)

**Keywords:** atrial fibrillation, atrioventricular node ablation, cardiac resynchronization therapy defibrillator (CRT-D), congestive heart failure, long-term mortality

## Abstract

**Objective:** In this study, we aimed to determine the most significant risk factors for 5-year mortality in patients with paroxysmal and persistent drug-refractory tachysystolic atrial fibrillation after undergoing atrioventricular node ablation (AVNA) in combination with the implantation of a permanent three-chamber pacemaker with an implantable cardioverter–defibrillator function (CRT-D). **Methods:** This prospective single-center cohort study included 101 patients with chronic heart failure (mean age 62 ± 15.5 years; 70.3% male) with paroxysmal or persistent drug-refractory atrial fibrillation who underwent atrioventricular node ablation and CRT-D implantation. All patients received optimal medical therapy before and after undergoing the procedure. Predictors of 5-year mortality were assessed using exploratory machine-learning methods, including random forest and Shapley additive explanations. **Results:** During 5-year follow-up, 13 cardiovascular deaths were recorded. Five key predictors of mortality were identified: left ventricular ejection fraction, 6 min walk distance, mean pulmonary artery pressure, systolic relaxation coefficient, and degree of mitral regurgitation. The exploratory predictive model showed high accuracy (92%) in terms of classifying the outcomes. **Conclusions:** Atrioventricular node ablation (AVNA) combined with CRT-D was associated with the observed long-term clinical outcomes observed in patients with drug-refractory tachysystolic atrial fibrillation. The exploratory machine learning analysis identified key mortality-associated factors, which may support future efforts in personalized risk stratification and hypothesis generation. The combination of AVNA and CRT-D was associated with the observed long-term outcomes in this real-world cohort.

## 1. Introduction

The combination of atrial fibrillation (AF) in patients with heart failure (HF) is a complex and multifaceted clinical process that significantly worsens outcomes and increases the economic burden on the global healthcare system. AF acts as an independent predictor of increased morbidity and mortality, increasing the risk of hospitalization in more than one-third of patients with HF [1]. Despite advances in pharmacological therapy and the introduction of modern medicines into widespread clinical practice, standard treatments often do not provide sufficient symptom control or quality of life improvement in patients with severe left ventricular dysfunction and uncontrolled ventricular contractions [2,3,4]. In such cases, depending on the left ventricular ejection fraction, it becomes necessary to implant resynchronizing devices without the function of a cardioverter defibrillator in patients with LVEF greater than 35% (CRT), and with the function of a cardioverter defibrillator with LVEF less than 35% (CPT-D), or interventional endovascular interventions, such as radiofrequency catheter ablation (RF). On occasion, a combined approach involving both methods is required to overcome the limitations of drug therapy and achieve a significant hemodynamic improvement [5].

Atrioventricular node ablation is of particular importance, which allows for 100% CRT functionality and significantly improves the clinical course of HF [6]. However, the idea of purposefully artificially destroying the natural conduction system of the heart raises ethical and practical doubts, since this intervention seems to contradict the principles of preserving the physiological function of the heart. Nevertheless, the combination of tachysystolic AF in patients with HF may cause a deterioration in hemodynamics and marked progression of HF.

Therefore, in this study, we present the analysis of our own long-term results of 5-year survival of patients with paroxysmal and persistent drug-refractory AF after AV ablation in combination with the implantation of a permanent three-chamber pacemaker with the function of a cardioverter defibrillator (CRT-D).

The purpose of this study is to determine the most significant risk factors for 5-year mortality in patients with paroxysmal and persistent drug-refractory tachysystolic AF after atrioventricular node ablation (AVNA) in combination with the implantation of a permanent three-chamber pacemaker with an implantable cardioverter–defibrillator function.

## 2. Materials and Methods

This prospective, single-center, real-world cohort study included 101 patients (71 men, 30 women, mean age—62 ± 15.5) with CHF who were treated from 2017 to 2019 at the Research Institute of Cardiology and Internal Diseases, Kazakhstan. The inclusion criteria were as follows: (1) functional class of CHF II-IV (NYHA); (2) paroxysmal or persistent drug-refractory atrial fibrillation. Fifteen patients had previously undergone conventional pulmonary vein ostial ablation; however, long-term maintenance of sinus rhythm was not achieved, resulting in progressive CHF with significant cardiac chamber dilatation, particularly of the left atrium (volume > 200 mL). In the remaining patients, pulmonary vein ablation was not considered due to advanced clinical deterioration with drug-refractory tachyarrhythmia and marked atrial and ventricular enlargement. The cohort also included patients with a history of valve surgery, for whom traditional pulmonary vein isolation was considered inappropriate.

All patients underwent ablation of the AV-node and were implanted with three-chamber pacemakers for CRT with a cardioverter defibrillator (CRT-D) function. The patients had no uncontrolled hypertension, hyperthyroidism, or hypothyroidism in the active phase, no heart defects or cardiomyopathies requiring surgical correction, and there were no data indicating a family history of channelopathy. Before undergoing ablation of the AV-node and implantation of the resynchronization device for at least ≥3 months and during the 5-year follow-up period, all patients received optimal drug therapy according to the current clinical guidelines.

Echocardiography (echocardiography) was performed using Philips EPIQ Elite (the Netherlands) according to the standard protocol. Left ventricular volumes (LVs) and LVEF were estimated using Simpson’s method in two-dimensional mode. Electrocardiography (ECG) was recorded according to the standard method in 12 leads at rest (duration and morphology of QRS complex were determined). Laboratory studies included the necessary tests in accordance with the current protocol for this pathology, including the dynamics of the natriuretic peptide (NT-proBNP) level. Clinical follow-up was performed over 5 years after CRT-D implantation with annual monitoring.

In this study, major adverse cardiac events (MACEs) were defined as deaths due to cardiovascular causes and other causes, which means cardiovascular mortality and total mortality, respectively.

**Figure 1:**
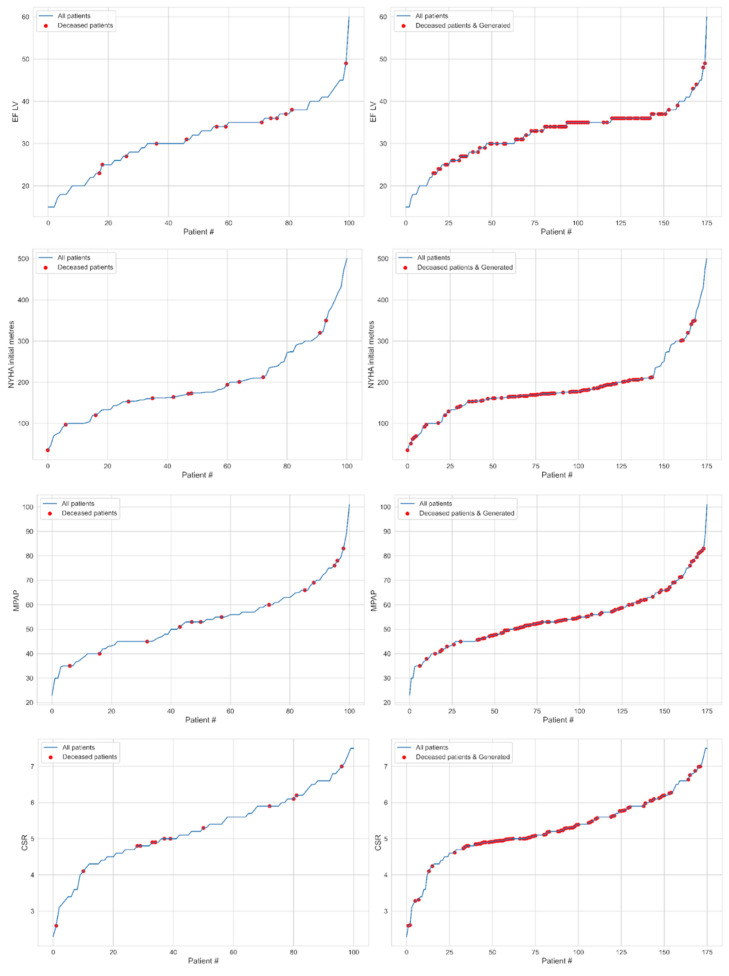
The patients (blue line) ordered for indicators EF LV (1st row), NYHA test (2nd row), MPAP (3rd row), CSR (4th row), and MR (5th row) marked the deceased cases (red dots) for real (**left**) and both real and synthetic (**right**) data.

Mathematical model description. The statistical data of 101 patients were not balanced according to the target indicator of the dead (13 patients) and the survivors (88 patients). Additional data from the minor class (dead class) were generated using the Stochastic SMOTE method based on the following algorithm (Figure 1):

1. Choose the patient who died during therapy.
2. Find the k = 5 neighbors of the same class using the Euclidean distance metric.
3. Generate synthetic data. Firstly, randomly select $x_{n}$ from k neighbors and create the new one as $x_{new}=x+r\left(x_{n}-x\right)$ between selected $x_{n}$ and initial x. Here, $r\in \left[0,1\right]$ is a random number from a uniform distribution.
4. Repeat steps 1–3 for the 75 generated “dead” patients.

Thus, the training set was supplemented with 75 synthetic “deceased” patients and 13 real patients from the deceased class.

For the indicators EF LV, NYHA test, MPAP, coefficient of systolic relaxation (CSR), and MR, the data (red dots) before the generation of patients (left) and after the generation (right) are detailed below.

Next, we used the random forest machine learning method to assess feature importance using the following algorithm:

1. We randomly divided all 176 data (real and synthetic) into trained (80%) and tested (20%) datasets.
2. The most optimal model hyperparameters were assessed for the available data using Gridsearch methods (direct overkill of combinations of parameters on the grid) and cross-validation of the trained data.
3. After performing a control check of the trained model with the obtained hyperparameters (training on 80% of a random sample of patients) and testing on 20%, the above accuracy was obtained. The sensitivity of the model was tested as follows:

(1) From full data (101 real patients and 75 generated patients), 20% of a random subset was obtained (36 patients) and the probability of death was predicted.
(2) Based on the predicted outcomes, a confusion matrix was constructed. The model successfully identified all deceased patients, while among the 18 predicted survivors, 15 were correctly classified as alive.

**Figure 2:**
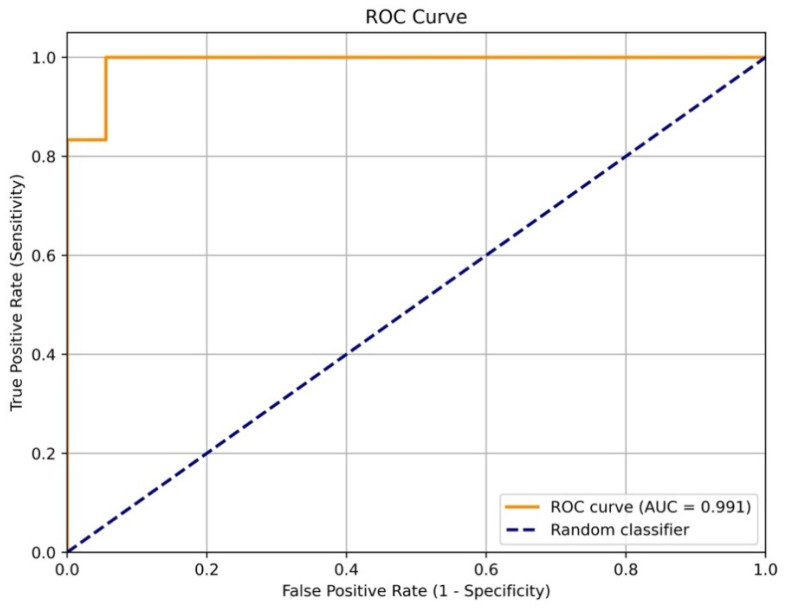
The ROC curve of model sensitivity to the 5-year fatal cardiovascular outcomes based on the test data (36 patients).

The performance of the model on the test sample (36 patients) is showcased in Figure 2, which shows that the model was wrong in only 8% of cases. For a more complete verification of the model’s accuracy, testing using an independent data sample is necessary.

Data were processed using the statistical software package SPSS v.26 (IBM, Armonk, NY, USA). Quantitative variables were presented as a median (25%; 75%); categorical variables were presented as percentages. The Mann–Whitney test, chi-square test, and correlation analysis were used to compare the groups. The level of significance was *p* < 0.05. Mathematical approaches from the field of machine learning were used to analyze the significance of the influence of the features selected for analysis on the 5-year survival rate after CRT. The first approach is the random forest method. The random forest model embeds itself a method for estimating the importance of the traits. The second method is based on the calculation of the values of the Shepley vector for each feature and comes from the field of the game theory, where each feature represents a “player” that contributes to the final prediction.

This study was performed according to the Declaration of Helsinki and the standards of Good Clinical Practice. All patients signed their informed consent to participate in this study. The study protocol was approved by the local ethics committee.

## 3. Results

In the period from 2017 to May 2019, 101 patients who met the criteria for inclusion in this study were enrolled in the Research Institute of Cardiology and Internal Diseases, Kazakhstan. The initial characteristics of this cohort are presented in Table 1.

Analysis of the initial data showed the following: the average left ventricular ejection fraction (LVEF) was 31.53 ± 7.7%, the average QRS values were 91.1 ± 15.5 ms, and the size of the left atrium was 4.95 ± 0.8 cm. Arterial hypertension was observed in the majority of patients, i.e., in 85 patients (84.2%). In total, 28 patients (27.7%) had a history of stroke, and 20 patients (19.8%) had a myocardial infarction. Diabetes mellitus was diagnosed in 13 patients (12.9%); all patients had tachysystolic atrial fibrillation, refractory to antiarrhythmic therapy, and all patients underwent implantation of a three-chamber EX with an implantable cardioverter–defibrillator function followed by radiofrequency ablation of the AV-node. The average body mass index of the patients was 29.8 ± 2.8 kg/m2.

Regarding the NYHA clinical classification of heart failure, FC II was registered in 9 patients (8.9%), FC III was registered in 70 patients (69.3%), and FC IV was registered in 22 patients (21.8%). The etiology of heart failure was predominantly ischemic in 65 cases (64.4%) and non-ischemic in 36 cases (36.6%).

Over the period of observation, 13 cases of death were recorded over five years, and all of these were due to cardiovascular complications. The drug treatment corresponded to modern clinical recommendations and included drugs for the treatment of heart failure and related diseases (Table 1).

When constructing a model of 5-year-old cardiovascular outcomes in multifactorial analysis, the following variables were included: age, patient body weight index, QRS width of the complex, the number of 6 min walk distance (meters), the degree of regurgitation on the mitral valve (reg), the diastolic relaxation coefficient, CSR, the “left ventricular ejection fraction” at the time of inclusion, the average pressure in the pulmonary artery, and scores for shocks (the clinical state assessment scale).

Since the initial data were not balanced according to the target indicator of the dead (13 patients) and survivors (88 patients), additional data were generated using the Stochastic SMOTE (Synthetic Minority Oversampling Technique) method. Further, to assess the importance of features, we used the random forest machine learning method (random forest method). The most optimal model hyperparameters were assessed for the available data using Gridsearch methods (direct overkill of combinations of parameters on the grid) and cross-packets and the following values were obtained: n_estimators = 150 (the number of trees), max depth = 7 (depth), min_samples_split = 2 (the minimum number of samples to divide the node), and min_samples_leaf = 1 (the minimum number of samples in the sheet). Control of a trained model with hyperparameters (80% of the random sample of patients) and testing with 20% (36 patients) meant that the accuracy of the model on the test selection was 92%, and the error matrix (confusion matrix) is presented in Table 2.

The presented matrix shows the following:

- All 18 dead patients were correctly identified;
- Out of 18 patients that were predicted to be living, this was true for 15 patients;
- The model “is playing it safe”, i.e., mistakenly predicting death for three surviving patients.

**Figure 3:**
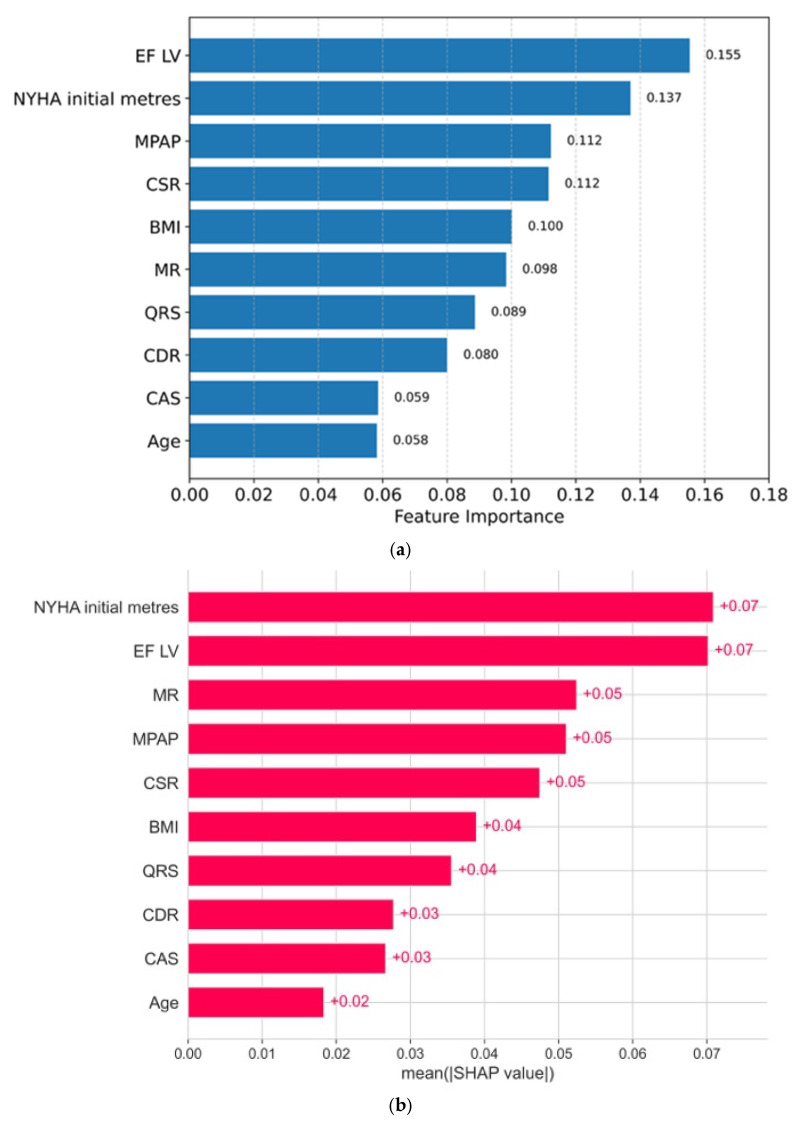
(**a**) The importance of signs: a random forest method. (**b**) The importance of signs: the SHAP method (average absolute SHAP indicators).

Thus, the obtained model corresponds to medical tasks, meaning that it does not miss mortal cases and is rarely reinsured. Since the random forest model itself integrates the method of assessing the importance of features, the contribution of each feature is presented in the diagram (all medical indicators are sorted by the degree of importance from greater to smaller; Figure 3a):

The second method that we used is based on the calculation of Shapley values for each feature and proceeds from the field of games theory, where each sign is a “player”, which contributes to the final forecast. According to the Shapley method, the predictions of the model with a contribution and without a contribution of each sign are evaluated. Figure 3b shows the signs and their importance in descending order, as obtained using the Shapley method.

Based on two different approaches, five of the most significant factors of 5-year mortality can be distinguished: EF LV (left ventricular ejection fraction), NYHA Initial meters (the value of the test of 6 min walking in meters), MPAP (mean pulmonary artery pressure), CSR, and MR (degree regurgitation on the mitral valve).

**Figure 4:**
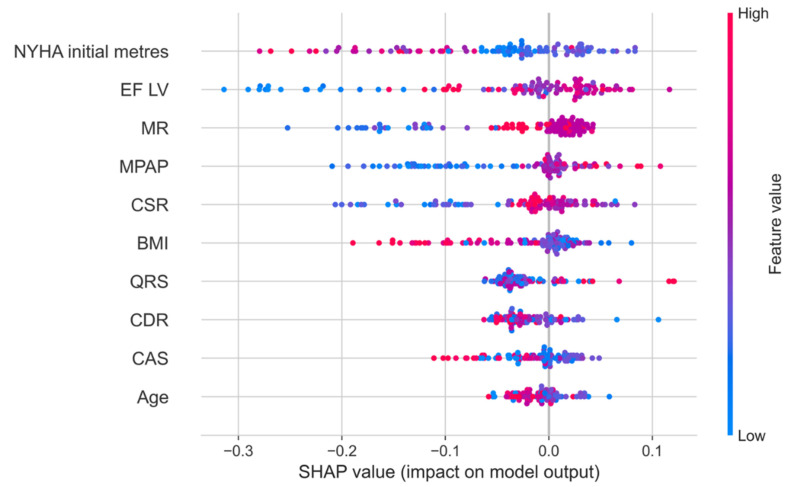
The dependence of the SHAP value of all medical indicators in 101 patients with a personalized assessment.

The Shapley method also allows for more detailed analysis of the contribution of each sign to the forecast of a particular individual. Figure 4 shows the dependence of the SHAP value of all medical indicators in 101 patients with their personalized assessment. Each point on the schedule corresponds to the characteristics of the indicator for a particular patient from the sample.

**Figure 5:**
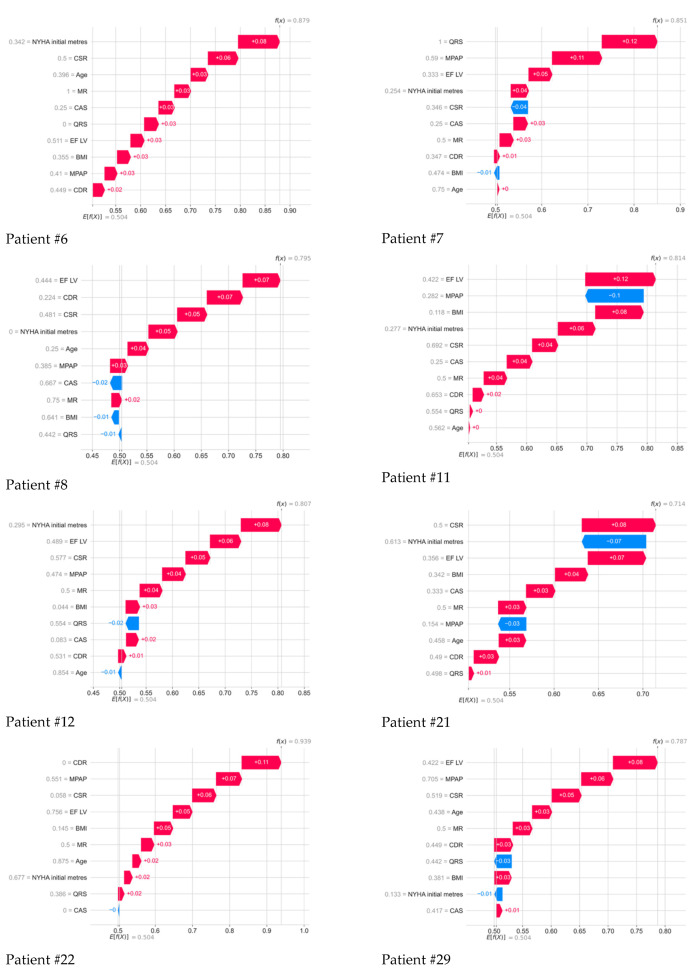

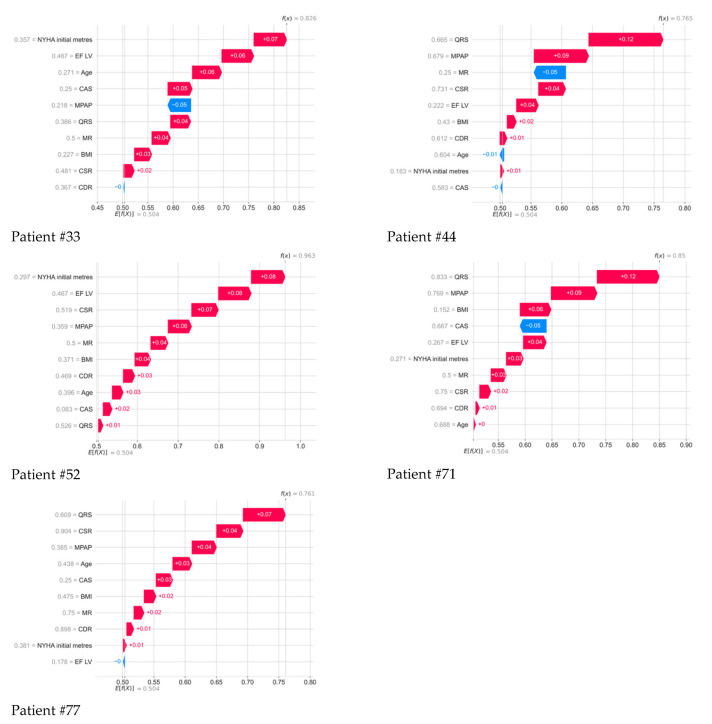
Contribution of all parameters to the 5-year outcome of each deceased patient, as determined by the Shapley method.

Figure 5 shows that higher indicators of the 6 min walking test and the body mass index, as well as lower indicators of mitral regurgitation, pressure in the pulmonary artery, and the systolic relaxation coefficient, indicate the best 5-year survival of the patients.

Further, the drawings show in detail the contribution of all parameters to the 5-year outcome of each deceased patient, determined using the method of whispers. Color numbers–signatures and their interpretation in the form of a colored strip is the SHAP meaning of each indicator: the higher their positive value, the greater contribution they make to the forecast of the death of a particular patient; the lower their negative value, the less contribution they make to a positive outcome. Next to each indicator, its normalized value from 0 to 1 for all indicators in the 101st patient is displayed.

## 4. Discussion

Currently, various approaches to managing patients with atrial fibrillation are used worldwide. Depending on the type of atrial fibrillation, the size of the cardiac chambers, and comorbidities, either a “rhythm control” or “rate control” strategy is considered. Despite the enormous advances in catheter treatment of atrial fibrillation, it should be remembered that in most patients, the likelihood of AF ablation failure remains high and may depend on the previously used technique (pulmonary vein isolation, “box-lesion”, mitral isthmus ablation, and CFAE ablation). Furthermore, the experience and opinion of the physician, the equipment and expertise of the center where the procedures are performed, and where multiple repeat ablation procedures may be part of the normal, sequential process of maintaining a “rhythm control” strategy, play a major role in long-term outcomes. Despite the modern AF treatment procedures performed by experienced physicians in leading global centers, patient quality of life and associated risks after unsuccessful AF ablation procedures still remain the cornerstone of this problem, leading to severe heart failure, decreased left ventricular ejection fraction, etc. Further research in the field of cardiac resynchronization therapy with defibrillator function and AV node ablation should focus on patient selection, intervention timing, and long-term follow-up of patient outcomes, including mortality, hospitalization, and heart failure manifestations, especially in those with low LVEF and drug-refractory AF. Therefore, since the 1980s, the technologies for treating AF have undergone many changes in recent decades, taking into account technological breakthroughs.

In 1982, Gallagher J. proposed the methodology of the percutaneous catheter ablation of the atrioventricular node for the first time [7]. At that time, it seemed almost contradictory that the deliberate destruction of such an important and physiologically functioning part of the heart—the main conducting node—could have practical value. However, in cases where a drug-refractory tachystolic form of atrial fibrillation (AF) is present, patients suffer significantly, which can lead to severe clinical consequences. The situation is especially unfavorable for the dysfunction of the left ventricle, when frequency control becomes critical to prevent the progression of heart failure. The strategy for creating a complete AV-block by means of catheter ablation of the AV-node can improve the control of the frequencies of ventricular contractions. It is especially important to emphasize that the radio frequency ablation of the AV-node does not restore the physiological function of the atrial and does not return the contractions to their normal rhythm, while the contractility of the ventricles completely depends on the implanted permanent pacemaker [5].

Additional technical considerations should be noted regarding the anatomical approach to atrioventricular node ablation. AV conduction ablation can be guided by targeting the penetration area of the conduction axis, identified by recording a His bundle potential. As highlighted in recent electrophysiological work, ablation of the His bundle may decrease the likelihood of a stable junctional escape rhythm after the procedure. Moreover, zero X-ray AV node ablation has emerged as a safe and effective option, providing technical advantages through electro-anatomical mapping and precise localization of His potentials without fluoroscopy, as demonstrated in recent zero-fluoroscopy series. Finally, the clinical significance of AV junction ablation in patients with atrial fibrillation and heart failure should also reference the APAF-CRT randomized trial, which showed that “Ablation + CRT” is superior to pharmacological rate control in reducing mortality and HF hospitalizations.

Before making a decision on ABA, it is necessary to work out all the possibilities of a medical reduction in heart rate. The choice of medications to control heart rate depends on symptoms, concomitant diseases, and possible side effects and interactions. Combination therapy with various drugs to control heart rate should be considered only in cases where it is necessary to achieve the target heart rate level, while careful monitoring should be ensured to avoid bradycardia. The combination of beta-blockers with verapamil or diltiazem should only be performed if emergency medical care is available 24/7.

Regarding drug therapy, all the possible potentially reversible risk factors and diseases that affect the onset and progression of AF should be taken into account. For example, a study based on the Danish registry (Frost et al.) showed that hyperthyroidism significantly increases the risk of atrial fibrillation (AF), especially in older men with concomitant cardiovascular diseases (such as coronary artery disease and heart failure). In particular, the risk increases with age, by 1.7 times for every ten years, which is consistent with the idea of AF as an age-associated disease [8].

Although the benefits of regular exercise in controlling cardiovascular risk factors are well established, little is known about the long-term cardiovascular effects of regular and extreme endurance sports such as running, cycling, rowing, swimming, etc. Recent evidence from small studies suggests that there is a link between regular, long-term practice of endurance sports and the development of atrial fibrillation. Some studies have shown that the size of the atria in athletes is larger than in the control group, and this is a predictor of the development of AF. Other suggested mechanisms are increased vagal tone and bradycardia, which affect the refractory period of the atria; however, this contributes rather than causes arrhythmia. In general, modern data indicate that there is a link between the practice of endurance sports and the development of AF [9]. To better understand this relationship, large-scale prolonged studies are needed, as well as a definition of possible thresholds for the intensity and duration of physical activity, at which the risk of developing AF can be minimal, while maintaining the cardioprotective effect of physical activity.

One large study showed that smoking is a significant risk factor for the development of AF; active smokers’ risk of AF is more than two times higher that of non-smokers, and its cessation can reduce the likelihood of AF [10]. The presence of so-called channelopathies, such as Brugada syndrome, can increase tachyarrhythmias, in particular ventricular [11]. Cardiomyopathies increase the risk of AF by about 1.5–2 times; AF is more common with hypertrophic and dilated CMP. The presence of AF in CMI is associated with higher mortality, a higher likelihood of hospitalization, the development of heart failure, and suffering a stroke. Catheter ablation in patients with cardiomyopathy and AF can reduce mortality [12].

The present study included patients with a drug-refractory tachystolic form of AF in combination with CHF; the included patients had no uncontrolled hypertension, hyperthyroidism, or hypothyroidism in the active phase, no heart defects or cardiomyopathies requiring surgical correction, and there were no data indicating a family history of channelopathy. Thus, the influence of these factors on the outcome of the disease was excluded. The strategy of the AVNA was combined with the implantation of a pacemaker in DDDR mode.

A number of researchers, such as Brignole M et al., have noted that the combination of ablation and implantation of the pacemaker in DDDR mode was more effective than drug treatment for symptoms control and quality of life improvement in patients with drug-refractory paroxysmal AF [13]. Similar results were also obtained in patients with chronic AF and clinically pronounced heart failure, which, after the ablation of the AV-node, implanted the pacemaker in VVIR mode [14].

In a number of randomized and incomplete studies that compare the effectiveness of catheter ablation of the AV-node in combination with the installation of a pacemaker and drug therapy [15,16,17], similar results were obtained. In a meta-analysis, which included 21 clinical examinations, an increase in tolerance to physical activity, fractions of the release of LV, and quality of life and a decrease in symptoms and the number of hospitalizations were revealed [18]. In another study, no significant differences were found in the endpoints of patients with a group of AV-nodes in combination with a pacemaker compared with a group of patients who did not undergo ablation of the AV-node, although the safety of both approaches is emphasized [19].

The “acceptance and stimulation” approach also has a side effect: the risk of sudden cardiac death (SCD). One of the first studies by Geelen P et al. [20] hypothesized this, but subsequent studies provided contradictory data: sudden death rates in the first year ranged from 0% to 9%, and multifactor analysis showed that the risk of SCD was higher in patients with impaired LV, severe heart failure, and ventricular arteritis [21]. One possible mechanism of SCD is the independent lengthening of the QT interval, which forms in such patients [21]. In recent years, stimulation frequency has gradually reduced from 90 beats per minute in the first 1–2 months after the procedure to a standard 60–70 beats per minute [22]. In addition, long-term right ventricle stimulation causes electrical and mechanical ventricular dyssynchrony in about half of patients, which also increases the risk of SCD, and three-chamber ECS reduces these risks [23].

In this article, we study the distant results and highlight the five most significant factors of 5-year mortality in patients with a paroxysmal and persistent drug-refractive tachysystolic form of AF after undergoing AV-node ablation in combination with implantation of a three-chamber pacemaker with an implantable cardioverter–defibrillator function: LV EF (left ventricular ejection fraction), NYHA Initial meters (value of the 6 min walk test in meters), MPPA (mean pressure in the pulmonary artery), CSR, and MR (degree of regurgitation on mitral valve). It is obvious that all these indicators characterize the expression of heart failure in patients with CHF and AF before the beginning of the combined surgical procedure.

We have expanded the rationale for CSR and MPAP as key predictors: CSR is associated with autonomic imbalance, cyclical hypoxia, and sympathetic overactivation, all of which worsen heart failure outcomes and promote arrhythmogenesis in AF + CHF patients. MPAP reflects pulmonary hypertension and increased right ventricular afterload, both recognized as strong prognostic markers in CHF that can significantly influence mortality and morbidity in AF patients.

Our approach now allows for a personalized evaluation of the CHF course using SHAP analysis, which quantifies the contribution of each predictor to an individual patient’s risk. This method can guide tailored follow-up, optimization of guideline-directed medical therapy (GDMT), device programming, and timing of interventions such as AVNA + CRT-D. Furthermore, SHAP-based subgroup analyses can help identify patients who are most likely to benefit from combined therapy, enabling more precise clinical management.

Modern optimal drug therapy can extend patients’ lives for, on average, eight years in patients with CHF III-IV on NYHA, but previous OMT work has not been evaluated in patients with CHF in combination with AF, so the forecast of such patients remains a subject of discussion [24]. In addition, the risks described in the literature relate to VVIR stimulation. Here, the risk of sudden death after the procedure was minimized by implanting a three-chamber ECS with an implantable cardioverter–defibrillator function and AV-node RFA [4].

In the presented work, all patients were implanted with three-chamber cardioverter defibrillators, so we did not observe cases of sudden cardiac death; all patients died from progression of CHF.

## 5. Conclusions

Radiofrequency catheter ablation of the atrioventricular node in combination with implantation of a permanent three-chamber pacemaker with an implantable cardioverter–defibrillator function represents a clinically meaningful intervention for improving outcomes in patients with severe drug-refractory tachysystolic AF and heart failure. Based on our findings, we suggest that implementing this approach to assess the risk of 5-year fatal cardiovascular outcomes may support personalized management of this complex patient population.

## Figures and Tables

**Table 1:** Clinical and functional characteristics of the included patients.

Total Number of Patients, *n*	*n* (101)
Mean age, years	62 ± 15.5
Men/women, *n* (%)	71 (70.3)/30(29.7)
Non-ischemic CHF, *n* (%)	36 (36.6)
CVD of ischemic genesis, *n* (%)	65 (64.4)
Diabetes mellitus, *n* (%)	13 (12.9)
Myocardial infarction, *n* (%)	20 (19.8)
Atrial fibrillation, *n* (%)	101 (100)
Atrioventricular node ablation, *n* (%)	101 (100)
Arterial hypertension, *n* (%)	85 (84.2)
Stroke history, *n* (%)	28 (27.7)
QRS complex duration, ms	91.1 ± 15.5
Left atrium, sm	4.95 ± 0.8
EF LV, %	31.53 ± 7.7
CSR LV, mL	528.8 ± 60.5
CDR LV, mL	647.2 ± 40.5
FC CHF NYHA II, *n* (%)	9 (8.9)
FC CHF NYHA III, *n* (%)	70 (69.3)
FC CHF NYHA IV, *n* (%)	22 (21.8)
ACE-i/ARA/ARNI, *n* (%)	101 (100)
Beta-adrenoblockers, *n* (%)	93 (92)
Ing CGLT 2, *n* (%)	84 (83.2)
Statins, *n* (%)	65 (64.4)
Antiplatelet agents, *n* (%)	65 (64.4)
Anticoagulants, *n* (%)	39 (38.6)
Antiarrhythmic drugs, *n* (%)	12 (11.9)
Loop diuretics, *n* (%)	32 (31.6)

**Table 2:** The ratio of predicted and actual fatal and nonfatal outcomes.

	Forecasting 0 (Alive)	Forecasting 1 (Death)
Reality 0 (alive)	15 patients	3 patients
Reality 1 (death)	0 patients	18 patients

## Data Availability

Data are available upon request from the corresponding author.

## Abbreviations

The following abbreviations are used in this manuscript:

ACE-i	angiotensin-converting enzyme inhibitors
AF	atrial fibrillation
ARA	angiotensin receptor antagonists
ARNI	angiotensin receptor–neprilysin inhibitors
AVJ	atrioventricular
CHF	chronic heart failure
CDR LV	left ventricular end-diastolic volume
CRT-D	cardiac resynchronization therapy with defibrillator
CSR	coefficient of systolic relaxation
CSR LV	left ventricular end-systolic volume
CVD	cardiovascular disease
EF	ejection fraction
EF LV	left ventricular ejection fraction
FC CHF NYHA	functional class of chronic heart failure according to New York Heart Association
MPAP	mean pulmonary artery pressure
NYHA	New York Heart Association
SGLT2	sodium–glucose cotransporter 2 inhibitors
SHAPs	Shapley additive explanations
